# Inhibition of androgen-independent prostate cancer cell growth is enhanced by combination therapy targeting Hedgehog and ErbB signalling

**DOI:** 10.1186/1475-2867-8-3

**Published:** 2008-03-18

**Authors:** Greg Shaw, David M Prowse

**Affiliations:** 1 Institute of Cancer, Bart's and The London School of Medicine and Dentistry, Queen Mary, University of London, London, UK; 2 Department of Urology, The Whittington Hospital, London, UK

## Abstract

**Background:**

Prostate cancer is a leading cause of male cancer specific mortality. When cure by radical prostatectomy is not possible the next line of prostate cancer treatment is androgen deprivation. However prolonged androgen deprivation often results in relapse and androgen-independent prostate cancer that is inevitably fatal despite optimal chemotherapy. The Hedgehog signalling pathway has recently been implicated in prostate cancer development and metastasis. EGFR or ErbB2 expression has been also correlated with androgen independence, shorter survival and metastasis.

**Results:**

We determined that the Hedgehog and ErbB signalling pathways are active in circulating tumour cells isolated from androgen-independent prostate cancer patients and in the androgen-independent prostate cancer cell line LNCaP C4-2B. As a basis for synergistic chemotherapy protocols combinations of the Hedgehog specific inhibitor cyclopamine and the ErbB signalling inhibitors gefitinib or lapatinib were tested in this study. Androgen-independent prostate cancer cell growth was inhibited by a SMO inhibitor (cyclopamine) which blocks Hedgehog signalling and by ErbB inhibitors (gefitinib and lapatinib). The isobologram and combination index method of Chou and Talalay was used to evaluate drug interactions. Synergistic antiproliferation effects were observed when the Hedgehog and ErbB inhibitors were combined.

**Conclusion:**

Androgen-independent prostate cancer cell proliferation was associated with activity of the Hedgehog and ErbB signalling pathways. Cyclopamine, gefitinib or lapatinib treatment significantly decreased the proliferation of androgen-independent prostate cancer cells. The Hedgehog pathway therefore represents a promising new therapeutic target in androgen-independent prostate cancer. Synergistic effects were observed when Hedgehog and ErbB inhibitors were used together. This study may have clinical implications for improving the treatment of advanced prostate cancer.

## Background

Prostate cancer is a leading cause of male cancer related deaths [1] and autopsy series have also found prostate carcinomas in the majority of men aged 60 to 70 years [2]. The incidence of prostate cancer diagnosis is also increasing as awareness improves, PSA measurement is performed more frequently and life expectancy increases [1]. Testicular factors were first linked to prostatic growth by John Hunter in 1786, although the endocrine nature of the relationship was not appreciated. Castration was subsequently shown by Charles Huggins in the 1940s to result in shrinkage of prostate cancer metastasis. Decreasing circulating testosterone with androgen deprivation therapy is currently used to treat metastatic prostate cancer and those cancers that are not suitable for attempts at cure with radiotherapy or surgery. This effectively shrinks androgen-dependent tumours, both in the prostate and at distant sites. However many men ultimately fail this therapy and continuous androgen deprivation usually leads to recurrent androgen-independent prostate cancer (AIPC)[3]. Once AIPC develops the median survival with the most effective therapeutic regimes is 20–24 months [4,5]. The high mortality rate associated with prostate cancer is therefore linked to the development of AIPC and the current lack of effective therapies. Developing new therapeutic approaches that target AIPC therefore has considerable potential for improving quality of life and survival of patients with advanced prostate cancer.

AIPC that arises as a consequence of androgen deprivation therapy may be due to increased activity of the androgen receptor (AR) or cell signalling pathways [6]. Growth factor signalling has been linked to ligand independent activity of the AR [6]. The ErbB receptor family are transmembranous receptors including EGFR, ErbB2, ErbB3 and ErbB4 which have intracellular tyrosine kinase domains. EGFR or ErbB2 expression has been correlated with androgen independence, shorter survival and metastasis [6-9]. Specific inhibitors of ErbB tyrosine kinase receptors have been developed. Gefitinib (Astra-Zeneca) is an EGFR receptor antagonist and lapatinib (Glaxo-Smithkline) has kinase inhibitor activity, inhibiting EGFR and ErbB2 activity. However their results in advanced prostate cancer trials to date have not been promising with the authors of one trial concluding that "gefitinib has minimal single-agent activity in AIPC" [10].

The Hedgehog pathway has also recently been implicated in prostate cancer development and metastasis [11]. Patched (PTCH) is the receptor for Hedgehog ligands (Sonic, Indian and Desert), which in the absence of Hedgehog inhibits Smoothened (SMO), a G protein coupled-like receptor. When Hedgehog binds to PTCH, SMO is disinhibited and initiates a signalling cascade that results in activation of GLI transcription factors and increased expression of target genes (including PTCH and GLI1). Inhibition of the Hedgehog pathway induces apoptosis and decreases invasiveness of prostate cancer cells [11]. Recent studies have shown a high prevalence of Hedgehog activity in high grade or metastatic prostate cancers [11,12], but the contribution of Hedgehog signalling to AIPC is unclear.

To clarify the role of ErbB and Hedgehog signalling in AIPC we determined that these pathways are active in both circulating tumour cells (CTC) isolated from patients with androgen-independent prostate cancer and in the androgen-independent prostate cancer cell line LNCaP C4-2B. The specific hedgehog pathway inhibitor cyclopamine and the ErbB pathway inhibitors gefitinib or lapatinib significantly decreased the proliferation of androgen-independent prostate cancer cells. A synergistic effect of Hedgehog and ErbB inhibitors on prostate cancer cell growth was also observed, consistent with both Hedgehog and ErbB signalling contributing to the proliferation of androgen-independent prostate cancer cells. The Hedgehog pathway therefore represents a promising new therapeutic target in androgen-independent prostate cancer.

## Results and discussion

**Figure 1:**
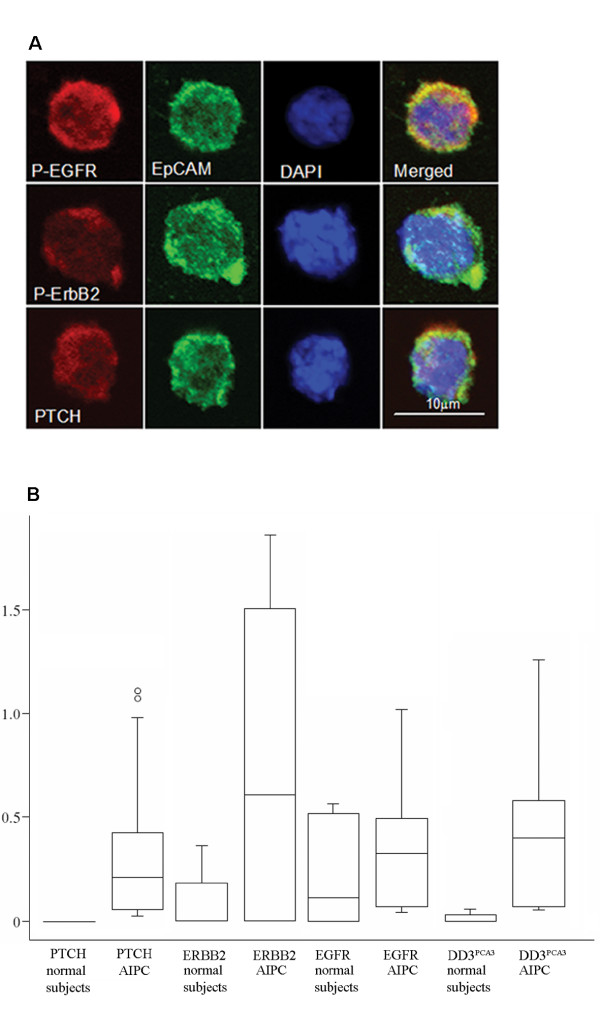
**Circulating tumour cells isolated from AIPC patients are EpCAM, PTCH, ErbB2 and EGFR positive.** (A) EpCAM, PTCH, ErbB2 and EGFR immunofluorescence in circulating tumour cells isolated from AIPC patients. (B) Relative PTCH, EGFR, ErbB2 and DD3^PCA3 ^RNA expression in circulating tumour cells isolated from normal subjects and androgen-independent prostate cancer patients.

To investigate the contribution of Hedgehog and ErbB pathways to AIPC we analysed the androgen-independent prostate cancer cell line LNCaP C4-2B and isolated CTC from fifteen patients with advanced prostate cancer who are on second line treatment having failed primary hormone therapy (with a rising PSA in the face of castrate serum testosterone levels) and are therefore androgen-independent, (clinical characteristics: age 53–89 years, median 76; last PSA 12–667 ng/ml, median 64; testosterone less than 0.7 nmol/L and duration of hormone therapy 1.2–13.9 years, median 4.4). The use of CTC is advantageous as biopsies are rarely performed on patients at this cancer stage and obtaining CTC is a low risk minimally invasive technique compared to biopsy. Immunofluorescence analysis showed that each prostate cancer patient sample contained more than 5 nucleated, EpCAM positive CTC, which has been associated with a poor prognosis in breast and prostate cancer [13,14]. No CTC were observed in the normal controls. CTC expressed PTCH, EGFR and ErbB2 protein (Figure 1A) and RNA (Figure 1B). A high background level of EGFR RNA expression was detected in the control samples enriched from healthy normal subjects. This expression of EGFR RNA by leukocytes carried over during the the CTC enrichment procedure was higher than previously reported [15]. In contrast, we observed very good discrimination between the normal subjects and the androgen-independent patient groups for ErbB2 (*p *< 0.005), PTCH (*p *< 0.005) and DD3^PCA3 ^(a prostate cancer specific RNA transcript DD3^PCA3^; *p *< 0.001) (Figure 1B), consistent with the Hedgehog and ErbB pathways contributing to AIPC.

**Figure 2:**
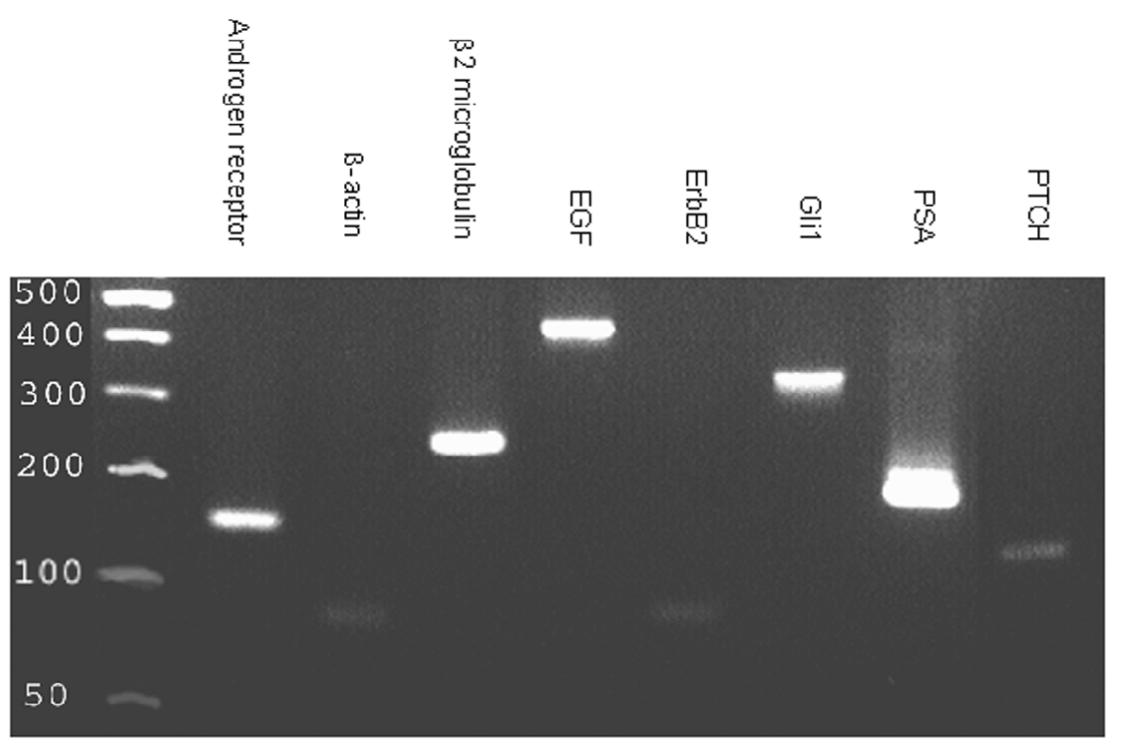
Expression of PTCH, GLI1, ErbB2, EGFR, PSA and AR RNA in LNCaP C4-2B cells.

As we have been unable to establish proliferating cultures of CTC for inhibitor and biochemical studies (Shaw and Prowse, unpublished), to further investigate the role of the Hedgehog and ErbB pathways in AIPC we have used the androgen-independent prostate cancer cell line LNCaP C4-2B. These cells were originally isolated and characterised following growth in castrated athymic mice of androgen-dependent LNCaP prostate cancer cells [16] from the site of bony metastasis (LNCaP C4-2B)[17]. Importantly, the growth of LNCaP C4-2B cells is not affected by withdrawal of androgens, confirming the androgen independence of these cells and these cells express androgen receptor and PSA (Figure 2) [17]. Hallmarks of the majority of prostate cancers *in vivo *[18,19] and characteristics not shared with other established prostate cancer cell lines such as PC3 and DU145 [16]. In addition, LNCaP C4-2B cells express a promiscuous form of the androgen receptor, having the most AR common substitution (T877A), which is repeatedly found in prostate cancer tissue specimens of patients with AIPC [20]. Like the CTCs, LNCaP C4-2B cells also express PTCH, EGFR and ErbB2 RNA (Figure 2).

**Figure 3:**
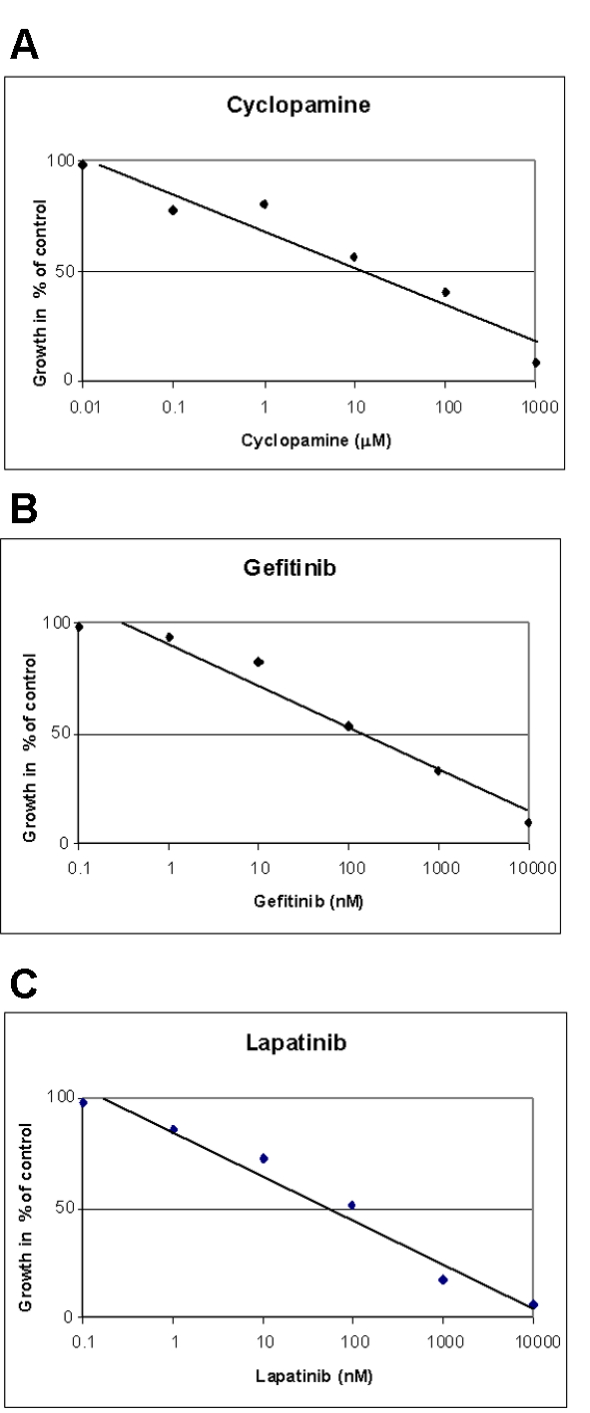
Effect of (A) cyclopamine, (B) gefitinib and (C) lapatinib on growth of androgen-independent prostate cancer cells.

**Figure 4:**
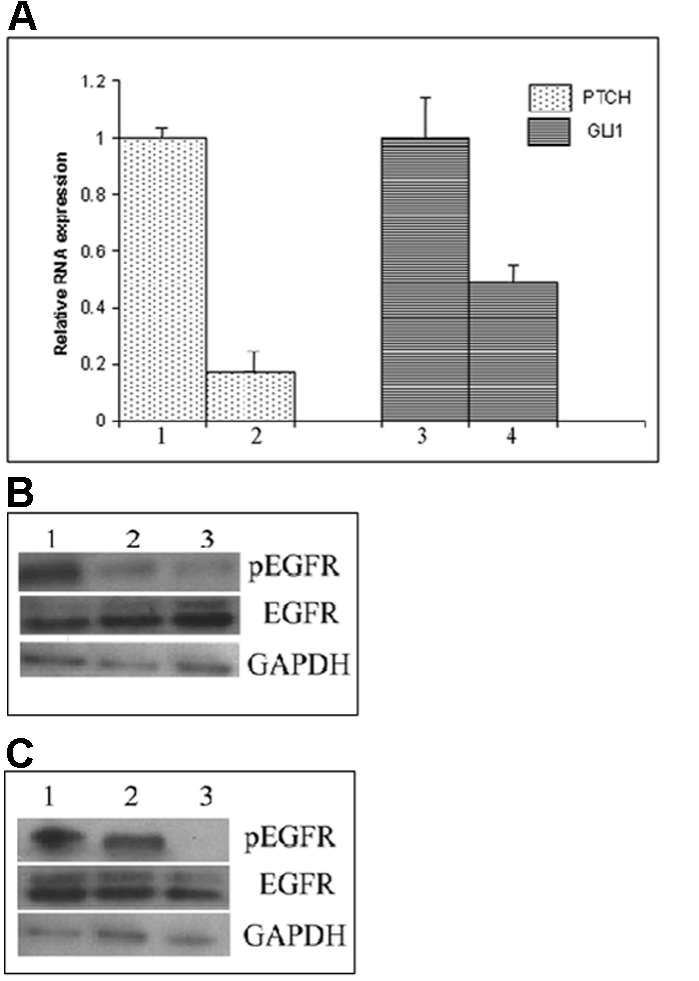
**(A) Cyclopamine inhibits expression of PTCH and GLI1 RNA in LNCaP C4-2B cells: control cells (lanes 1 and 3); 24 hrs 14 nM cyclopamine treatment (lanes 2 and 4).** Relative expression determined by QPCR (error bars show standard deviation). (B) Immunoblot showing inhibitory effect of 168 nM gefitinib on EGFR phosphorylation following EGF treatment in AIPC cells (lane 1: control; lane 2: 24 hr gefitinib; lane 3: 48 hr gefitinib). (C) Immunoblot showing inhibitory effect of 102 nM lapatinib on EGFR phosphorylation following EGF treatment in AIPC cells (lane 1: control; lane 2: 24 hr lapatinib; lane 3: 48 hr lapatinib).

To determine the importance of the Hedgehog and ErbB pathways to AIPC cell growth we treated LNCaP C4-2B cells with specific inhibitors to cyclopamine (SMO inhibitor) which blocks Hedgehog signalling, gefitinib (EGFR inhibitor) and lapatinib (EGFR and ErbB2 inhibitors), either singularly or in combination. The growth of LNCaP C4-2B cells in androgen free medium was significantly reduced by treatment with the Hedgehog pathway inhibitor cyclopamine, the EGFR inhibitor gefitinib and the EGFR and ErbB2 inhibitor lapatinib. The effects were dose dependent. Using cyclopamine between 0.0014–1 mM, gefitinib at 0.017–10 μM and lapatinib at 0.01–10 μM there was minimal affect at the lowest dose for each inhibitor and significantly greater inhibition at higher concentrations (Figure 3). Calculation of the drug concentration (IC_50_) producing the median effect of 50% growth inhibition on the LNCaP C4-2B cell line in androgen free medium was performed from the dose response curves for each drug (cyclopamine 14 μM; gefitinib 168 nM; lapatinib 102 nM; Figure 3), and were similar to those reported in the literature [21-23]. The PTCH receptor and GLI1 transcription factor are both constituents of the hedgehog pathway which are also regulated by Hedgehog signalling. Application of 14 μM cyclopamine for 24 hours to androgen-independent LNCaP C4-2B cells resulted in decreased expression of PTCH and GLI1 (Figure 4A), consistent with cyclopamine inhibiting SMO and Hedgehog signalling activity. The ErbB inhibitors gefitinib (168 nM) and lapatinib (102 nM) also inhibited EGF induced autophophorylation of the EGFR in LNCaP C4-2B cells (Figure 4B–C).

**Figure 5:**
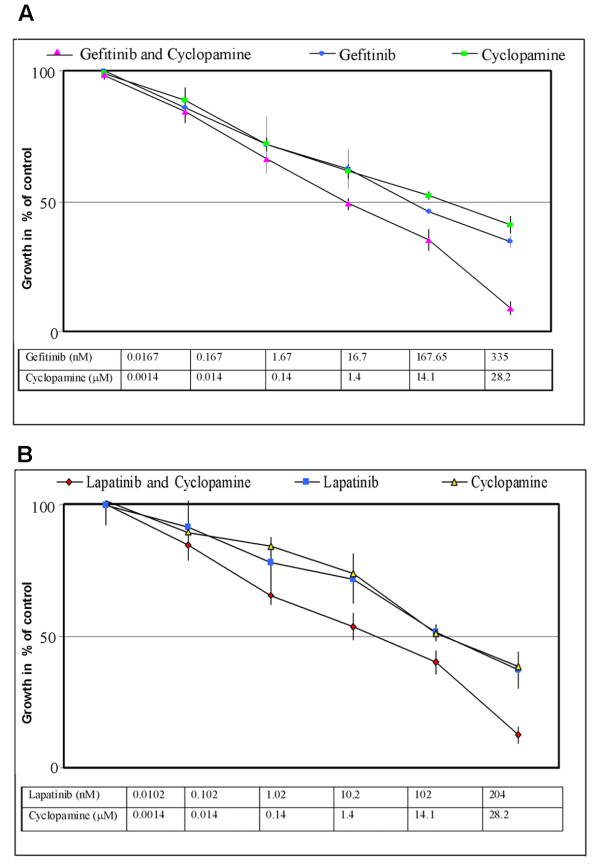
Concentration effect curves of (A) single agent cyclopamine and gefitinib and their combinations, (B) single agent cyclopamine and lapatinib and their combinations.

**Figure 6:**
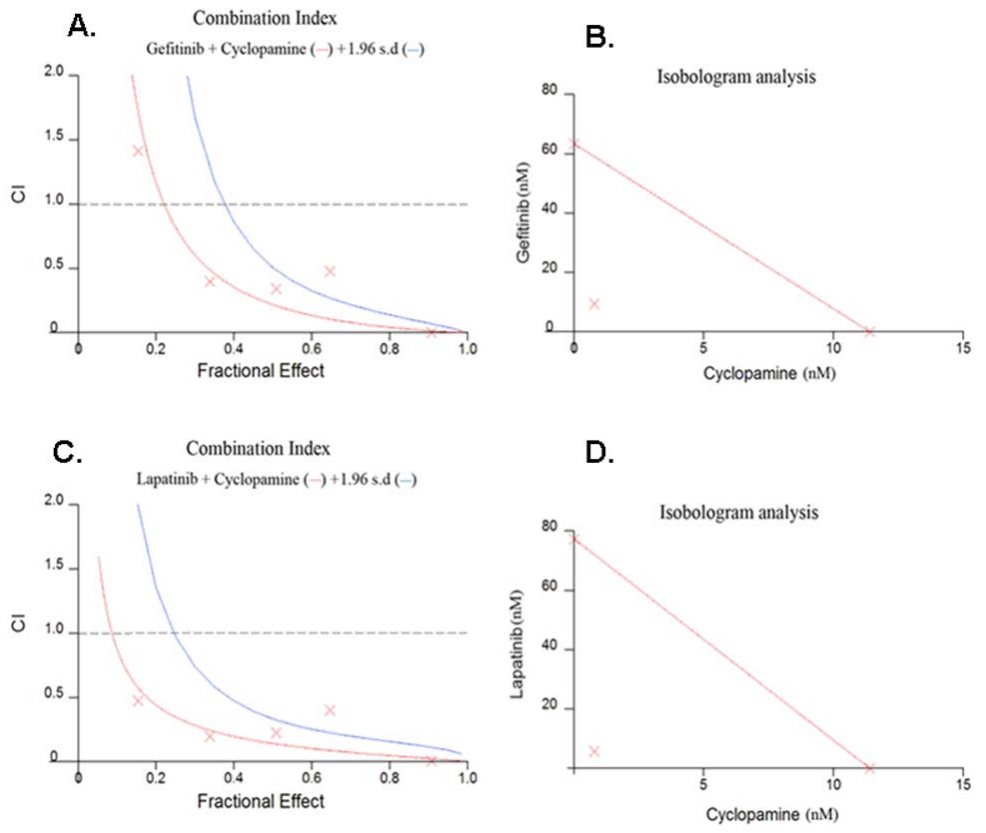
**Analysis of cyclopamine and (A-B) gefitinib or (C-D) lapatinib in LNCaP C4-2B cells.** (A and C) combination index plot for the drug combinations. (B and D) Isobologram for the combination of gefitinib or (C-D) lapatinib and cyclopamine for effect level (F_a _= 0.5). Note the combination data points fall on the lower left of the hypotenuse for F_a _= 0.5 (shown), F_a _= 0.75 and F_a _= 0.9 indicating synergism.

In order to establish whether the combined effects of Hedgehog and ErbB inhibitors were synergistic the isobologram and combination index (CI) was calculated according to the Chou and Talalay median effect principal [24]. Inhibitors were applied to androgen-independent LNCaP C4-2B cells at concentrations relative to their respective IC_50 _values keeping the ratio of one drug to the other constant. For each drug combination the MTT assays were carried out in three separate experiments and the relative growth rates calculated in comparison with LNCaP C4-2B cells cultured in androgen free medium in the absence of any cytotoxic drugs. The Hedgehog inhibitor cyclopamine as single agent or in combination with the ErbB inhibitors gefitinib or lapatinib inhibited the growth of LNCaP C4-2B cells. Figure 5A shows the dose response curve for cyclopamine and gefitinib applied alone and in combination and Figure 5B shows the dose response curve for cyclopamine and lapatinib applied alone and in combination. Figure 6 shows the combination effect plots and isobolograms for the inhibitor combinations. Table 1 shows the combination index for treating androgen-independent LNCaP C4-2B cells with inhibitor combinations, with values below 0.9 indicating synergism and above 1.1 antagonism. Strong synergistic effects resulted from the combination of cyclopamine with gefitinib or lapatinib.

**Table 1:** Dose-effect relationships of drug combinations in human androgen-independent prostate cancer cell line LNCaP C4-2B: Gefitinib (Ge), Lapatinib (La) and Cyclopamine (Cy).

Treatment	Parameters			CI value at			Range	Significance
						
	D_m_	m	r	ED_50_	ED_75_	ED_90_		
Ge+Cy (12:1)	9.5	0.44	0.94	0.22	0.06	0.02	0.1–0.3	strong synergism
La+Cy (7:1)	5.8	0.44	0.94	0.14	0.06	0.03	0.1–0.3	strong synergism

This is consistent with the antiproliferative results recently reported following treatment with cyclopamine or gefitinib of androgen dependent LNCaP-C33 cells, the spontaneously arising androgen-independent LNCaP subline C81 and androgen independent DU145 and PC3 cells [21,25]. Importantly, combined cyclopamine and gefitinib treatment was also found to cause a high rate of inhibition of proliferation [21] and a significant increase in apoptotic death of androgen-independent LNCaP-C81, DU145 and PC3 cells, although androgen dependent LNCaP-C33 cells were less responsive to these agents [21]. Our CTC analysis is also consistent with reports that specimens from advanced prostate cancer have higher levels of SHH, PTCH-1 and GLI-1 as compared to samples from localized PC and normal tissues or benign PrE cells [11,21,26].

The synergy between cyclopamine and gefitinib or lapatinib may occur because of interactions between the Hedgehog and ErbB pathways, consistent with EGF signalling selectively enhancing Hedgehog activity [27] and cyclopamine treatment of PC3 cells causing downregulation of EGFR expression [21]. Gefitinib has also been reported to inhibit the activity of the androgen receptor, enhancing its anti-proliferative affect [28]. Hedgehog and ErbB signalling may also contribute to prostate cancer metastatsis as we have found expression of these genes in CTC isolated from the peripheral blood of AIPC patients, gefitinib treatment has been reported to inhibit EGF induced invasion of prostate cancer cells [29] and Hedgehog signalling has also been linked to metastasis [11,12]. Combination chemotherapy targeting these signalling pathways therefore also has the potential to be beneficial in metastatic prostate cancer.

Our findings are consistent with Hedgehog and ErbB being of therapeutic relevance to the management of prostate cancer. Hedgehog signalling may be an important new target in metastatic AIPC. Although, at present, there is no clinically available treatment that specifically targets the Hedgehog signalling pathway. The SMO inhibitor cyclopamine, which we show can be used to inhibit AIPC cell proliferation, along with other Hedgehog signalling targeting compounds are currently being developed (Curis, USA) and a Phase I clinical trial of a systemically administered small molecule Hedgehog antagonist initiated (Genentech, USA). In addition, as significant clinical improvements have not been reported using ErbB signalling inhibitors alone (Herceptin or Gefitinib) in phase II clinical trials for advanced prostate cancer [10,30]. Combination therapy targeting both Hedgehog and ErbB signalling may enable enhanced anticancer efficacy with no greater toxicity, thus improving the treatment of advanced prostate cancer.

## Conclusion

Our results suggest that the Hedgehog and ErbB signalling may play an important role in the proliferation of androgen-independent prostate cancer cells. As we observed expression of PTCH, GLI1, EGFR and ErbB2 in AIPC cells and that inhibitors of these signalling pathways in combination had synergistic anti-proliferative effects. The Hedgehog pathway therefore represents a potential new therapeutic target in advanced prostate cancer and combination therapy against Hedgehog and ErbB pathways could also be considered.

## Methods

### Cell and tissue culture

Androgen-dependent LNCaP cells were obtained from the American Type Culture Collection (ATCC). Androgen-independent LNCaP C4-2B cells were purchased from Viromed Laboratories. Cells were grown at 37°C in RPMI media without phenol red (Sigma) supplemented with 10% (v/v) fetal bovine serum (Invitrogen) or 10% (v/v) charcoal stripped FBS (Hyclone), 2.4 mM glutamine (Sigma), 1% (v/v) pyruvate (Sigma), penicillin and streptomycin (50 U and 50 μg/ml) (Invitrogen). Cells were treated with EGF (Sigma), cyclopamine (Sigma), gefitinib (AstraZenica) and lapatinib (Glaxo-Smithkline) as detailed.

### Isolation of circulating tumour cells

Peripheral blood samples were obtained from 15 patients (anonymised for confidentiality according to the format ABC 12) and 5 normal male subjects (named NORM 1–5, all under 40 years of age with no significant intercurrent illness). Two 10 ml peripheral blood samples were collected in EDTA coated tubes (Greiner), kept on ice and processed within three hours of collection. Samples were processed through Oncoquick (Greiner) tubes, as per the manufacturer's instructions. In order to further purify the CTC the Oncoquick enriched samples were depleted of CD45 positive cells and positively selected for EpCAM expressing cells using AutoMACS™ (Miltenyi biotech). The study was fully approved by the Local Research Ethics Committee (reference number 05/Q0602/48).

### RNA isolation, amplification, reverse transcription and quantitative PCR

RNA was isolated from the samples using the RNeasy Mini kit (Qiagen). cDNA was synthesized following DNAse treatment by reverse transcription using Superscript III (Invitrogen) with oligo-dT priming. QPCR was performed using Platinum SYBR^®^Green qPCR Supermix-UDG (Invitrogen) using the Opticon DNA Engine 2™. Primers used are detailed in Table 2. The relative expression of β_2_-microglobulin was used as an internal control.

**Table 2:** Oligonucleotides used for PCR amplification

Gene	Sequence 5'-3'	Size of product (Base Pairs)
AR-f	GACGCTTCTACCAGCTCACC	60
AR-r	GAAAGGATCTTGGGCACTTG	
β-actin-f	GATGAGATTGGCATGGCTTT	60
β-actin-r	CACCTTCACCGTTCCAGTTT	
DD3^PCA3^	GGTGGGAAGGACCTGATGATAG	58
DD3^PCA3^	GGGCGAGGCTCATCGAT	
ErbB2-f	CCTCTGACGTCCATCGTCTC	100
ErbB2-r	CGGATCTTCTGCTGCCGTCG	
EGFR-f	CTTCTTGCAGCGATACAGCTC	439
EGFR-r	ATGCTCCAATAAATTCACTGC	
GLI1-f	GAAGACCTCTCCAGCTTGGA	306
GLI1-r	GGCTGACAGTATAGGCAGAG	
GREX-f*	GAGTACCTCTGGAGGACAGA	281
GRINTRON-r*	ATGTCCATTCTTAAGAAACAGGA	
PSA-f	GGTGACCAAGTTCATGCTGTG	194
PSA-r	GTGTCCTTGATCCACTTCCG	
PTCH-1-f	CTCCCAAGCAAATGTACGAGCA	150
PTCH-1-r	TGAGTGGAGTTCTGTGCGACAC	
β2-microglobulin-f	TGAATTCGTATGTGTCTGGGT	247
β2-microglobulin-r	CCTCCATGATGCTGCTTACAT	

* GREX/GRINTRON = glucocorticoid receptor intron genomic DNA control. Sequence for GRINTRON crosses boundary of glucocorticoid receptor gene intron and exon. No product is formed unless genomic DNA is present.

### Antibodies and immunoblotting

Primary antibodies used were as follows: EGFR (#2232 Cell Signalling); Phospho-EGFR (#2231 Cell Signalling); Phospho-ErbB2 (#2241 Cell Signalling); Patched (#sc-6149 Santa Cruz Biotechnology); EpCAM (#ab20160 Abcam) and GAPDH (# ab9485 Abcam). For immunofluoresence staining CTCs were air dried onto slides, fixed with methanol and incubated with primary antibodies (1:400 phospho-EGFR; 1:250 Phospho-ErbB2; 1:250 EpCAM; 1:200 Patched) overnight in blocking buffer and detected using appropriate secondary antibodies. Nuclear counterstaining was performed using DAPI. Stained preparations were analysed on a Zeiss 510 confocal microscope. For western blotting cultured cells at 70–80% confluence were washed twice with PBS and lysed in RIPA buffer containing Sigma Phosphatase Inhibitor Cocktails I & II and Roche Protease Inhibitor Complete EDTA-free. Lysates were cleared by centrifugation at 16,000 × g for 10 mins. 20 ug of each lysate was boiled for 10 mins in Invitrogen sample buffer with antioxidant and resolved using the Invitrogen NuPage system on a 10% Bis-Tris gel with MOPS buffer, then transferred to Amersham Hybond-C extra membrane. The membranes were blocked for 30 mins in 5% milk or 5% BSA (GAPDH) in TBS-T. Primary antibodies (1:1000 pEGFR and pEGFR; 1:5000 GAPDH) were incubated overnight at 4°C with blocking and visualised using an appropriate HRP-conjugated secondary antibody using enhanced chemiluminescence (ECL, Amersham).

### MTT assay for cell proliferation

The MTT [3-(4,5-dimethylthiazol-2-yl)-2, 5-diphenyltetrazolium bromide] assay is based on the ability of a mitochondrial dehydrogenase enzyme from viable cells to cleave the tetrazolium rings of the pale yellow MTT and form dark blue formazan crystals which are largely impermeable to cell membranes, thus resulting in its accumulation within viable cells. The colour can then be quantified using a simple colorimetric assay. Cells were seeded onto 96 well plates at 2–4 × 10^4^/well in 200 μl media and synchronized in G_0 _(quiescent) phase of the growth cycle by culture in serum free medium. After 12 hours this serum free medium was replaced with 10% Fetal Bovine Serum in RPMI containing inhibitors as detailed. After 48 hours 20 μl of the 5 mg/ml MTT substrate was added to each well. Plates were returned to the incubator and left in the dark for 1 hour. After the incubation period, MTT substrate/medium was gently removed from each well and 200 μl of DMSO were added to each well to dissolve the MTT formazan crystals and absorbance measured spectrophotometrically at a wavelength of 570 nm. Blank control values were then subtracted from the 570 nm values and relative growth rates were calculated. All experiments carried out using MTT assay were carried out on three separate occasions.

### Determination of IC_50 _concentration for cytotoxic drugs

Androgen-independent LNCaP C4-2B cells growing in androgen free medium were harvested by trypsinisation, washed and seeded at 30,000 per well in 96 well plates and left to attach overnight. The IC_50 _doses were determined by exposing cells to various concentrations of the drugs 10^-7^–10^-3^M for 72 hours. The medium with drug was aspirated and the MTT assay described above was performed. The IC_50 _was defined as the concentration of drug at which there was a 50% less growth when compared to control cells. Each experiment was performed in triplicate.

### Median effect analysis

The isobologram and combination index (CI) were calculated according to the Chou and Talalay median effect principal [24] using Calcusyn software (Biosoft). The drugs were applied at a fixed ratio of the IC_50 _across a range of activities and viability was evaluated using the MTT assay at each dosage. Data from cell viability assay were expressed as the fraction of cells inhibited by drug treatments compared with untreated cells. Interaction between pairs of drugs was determined using the Calcusyn computed isolobogram and combination index (CI). The isobologram is a graphical representation of the interaction between two drugs and is formed by plotting the individual drug doses required to achieve a single agent effect on their respective x and y axes, a line connecting the two points is drawn and the concentrations of the two drugs used in combination to achieve the same effect are plotted on the isobologram. Combination data points that fall on the line represent an additive interaction, whereas points above or below represent antagonism or synergy respectively. The CI analysis is similar to the isobologram provides qualitative information on the drug interaction and a numerical CI value is calculated based on the following equation: CI = (D)1/(Dx)1 + (D)2/(Dx)2 + (D)1(D)2/(Dx)1(Dx)2, where (D)1 and (D)2 are the doses of drug 1 and drug 2 that have x% effect when used in combination, and (Dx)1 and (Dx)2 are the doses of drug 1 and drug 2 that have the same x% effect when used alone. The CI indicates synergism when <0.9, antagonism when >1.1 and additivity when 0.9–1.1. The Calcusyn software also calculates the median effect dose (*D*_*m*_) of each combination, shape of the dose-effect curve (*m*) and linear correlation coefficient of the median effect plot (*r*) indicating conformity of date.

## Competing interests

The author(s) declare that they have no competing interests.

## Authors' contributions

GS: Experiments (including cell culture and preparation, PCR, drug treatments and synergy calculations). DMP: Experimental design, synergy calculations, manuscript preparation. All authors have read and approved the final manuscript.
